# Genetic insights into the crude protein and fiber content of ramie leaves

**DOI:** 10.3389/fpls.2022.969820

**Published:** 2022-10-04

**Authors:** Zhiyong Liu, Zheng Zeng, Xiai Yang, Siyuan Zhu, Touming Liu, Yanzhou Wang

**Affiliations:** ^1^College of Agriculture, Yangtze University, Jingzhou, China; ^2^Institute of Bast Fiber Crops, Chinese Academy of Agricultural Sciences, Changsha, China; ^3^College of Horticulture and Plant Protection, Yangzhou University, Yangzhou, China

**Keywords:** ramie, crude protein content, crude fiber content, quantitative trait locus, MYB gene

## Abstract

Ramie (*Boehmeria nivea* L.) is a perennial plant with vigorously vegetative growth and high nutritive value that is an excellent source of green feed in China. Crude protein and fiber content are the most important traits associated with ramie forage quality; however, their genetic basis remains largely unknown. In this study, we investigated the genetic architecture of these two traits using an F_2_ population derived from cultivated Zhongsizhu 1 (ZSZ1) and wild *Boehmeria nivea* var. *tenacissima* (tenacissima). Linkage mapping identified eight quantitative trait loci (QTLs) in crude fiber and one QTL in crude protein. Of these, five were further validated by association analysis. Then, two major QTLs for crude fiber content, *CF7* and *CF13*, were further identified using bulked segregant analysis (BSA) sequencing, and their exact physical intervals were determined *via* genotype analysis of F_2_ progenies with extremely low crude fiber content. In total, 10 genes in the *CF7* and *CF13* regions showed differential expression in ZSZ1 and tenacissima leaves, including an MYB gene *whole_GLEAN_10016511* from the *CF13* region. Wide variation was observed in the promoter regions of *whole_GLEAN_10016511*, likely responsible for its downregulated expression in tenacissima. Interestingly, more fiber cells were observed in *Arabidopsis* with overexpression of *whole_GLEAN_10016511*, indicating that the downregulated expression of this gene could have an association with the relatively low fiber content in wild tenacissima. These results provided evidence that *whole_GLEAN_10016511* is a logical candidate for *CF13*. This study provides important insights into the genetic basis underlying ramie crude protein and fiber content, and it presents genetic loci for improving the forage quality of ramie using marker-assisted selection.

## Introduction

Ramie is a traditional, natural fiber crop with a cultivation history of over 4,700 years in China (Li, 1970). It is a perennial plant that is usually harvested by cutting mature shoots without destroying the roots, thereby allowing the root system to continuously develop in the soil (Subandi, 2012). Ramie exhibits vigorous, vegetative growth. When it is watered regularly, it can be harvested 6–8 times per year, resulting in 126 tons of fresh biomass per hectare per year (Rehman et al., 2019). In addition, previous studies have shown that young shoots and leaves contain 16.4% crude protein, 25% digestible fiber, 31.8 g/kg calcium, 18.7 g/kg potassium, and ample amounts of lysine, methionine, carotenoids, and riboflavin. Ramie also has high metabolic energy levels (6.4 MJ/kg dry matter; INFIC, 1978), indicating high nutritive value. As the nutritional value of ramie is similar to that of alfalfa (*Medicago sativa*), ramie is an excellent nutritive source of green feed in China and palatable to all classes of farm animals (Rehman et al., 2019). Previous research on cattle, sheep, pigs, horses, and poultry has highlighted the importance of ramie as a key nutritional source in the form of green foliage (Pérez et al., 2013).

Crude protein and fiber are two of the most important traits associated with ramie forage quality. In the past decade, there has been considerable progress in genetic and genomic research on ramie. In total, thousands of simple sequence repeat markers (SSRs) have been developed based on RNA sequencing transcripts (Liu et al., 2013; Chen et al., 2016). Based on these markers, the first genetic SSR map was developed for ramie (Liu et al., 2014), including 16 SSRs associated with fiber yield traits (Luan et al., 2017). The rapid development of next-generation sequencing technology has recently led to the identification of large-scale single-nucleotide polymorphisms (SNPs) in ramie, resulting in several high-density genetic maps (Liu et al., 2017; Zeng et al., 2019). Consequently, the genetic architecture of many agronomic traits, such as fiber yield, quality, and flowering time, has been systematically characterized using linkage mapping and genome-wide association mapping (Zhu et al., 2016; Liu et al., 2017; Chen et al., 2018, 2019; Zeng et al., 2019, 2022; Huang et al., 2021; Wang et al., 2021), and several candidates across various loci have been documented (Liu et al., 2017; Wang et al., 2021; Zeng et al., 2022). Recently, ramie feeding traits have also been researched, and the genetic basis of six forage yield-related traits was characterized, resulting in 78 significant association signals from 43 genomic regions (Bai et al., 2022). However, in terms of forage quality traits, specifically the crude protein and fiber content, the genetic basis remains largely unknown, limiting our knowledge of the genetic potential of ramie. In a previous study, we developed an F_2_ population consisting of 111 progenies derived from cultivated Zhongsizhu 1 (ZSZ1) and the wild species *B. nivea* var. *tenacissima* (hereafter, tenacissima). Based on this population, a high-density genetic map with 1,085 SNPs was constructed, with a total length of 2,118.8 cM (Wang et al., 2019). In this study, we explored the genetic architecture of crude protein and fiber content based on the F_2_ population; furthermore, a major quantitative trait locus (QTL) of the crude fiber content, *CF13*, was analyzed in-depth to ascertain its suitability as a candidate.

## Results

### Phenotypic variation in the F_2_ population and parents

**FIGURE 1:**
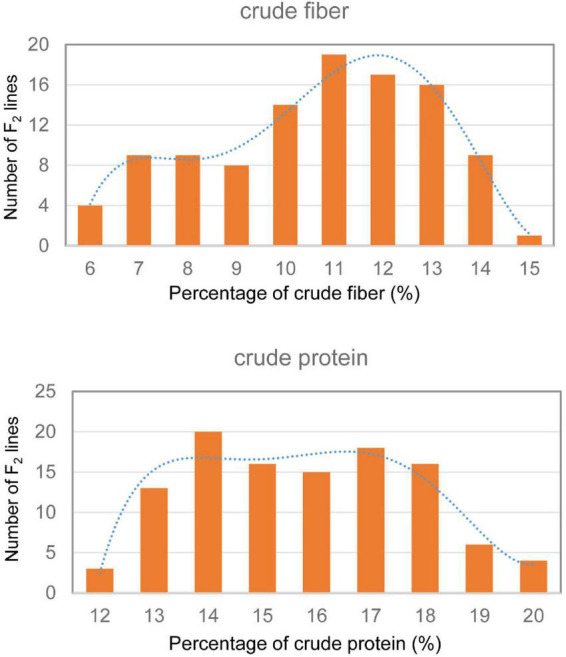
Frequency distribution of crude protein and fiber content in the F_2_ population derived from cultivated ZSZ1 and wild tenacissima. Blue dashed line indicates the frequency distribution trend. The *x*-axis indicates the percentage (%) of crude protein and fiber, and the *y*-axis indicates the number of F_2_ lines.

Only 6.28% crude fiber and 15.63% crude protein were observed in the dry leaves of the parent tenacissima, whereas 14.14% crude fiber and 19.42% crude protein were observed in ZSZ1, indicating a considerable difference between each trait in the parents. Within the population, the percentage of crude fiber in the dry leaves ranged from 5.23 to 14.48%, with a mean of 10.17%, whereas the percentage of crude protein in the dry leaves ranged from 11.25 to 19.35% (15.30 ± 1.99%); this indicated a wide variation in the two traits associated with forage quality, in this population. Correlation analysis revealed that the phenotypic values of these traits were not correlated in this population (*p* > 0.05). Furthermore, the crude fiber and protein content followed a skewed normal distribution (Figure 1). These results indicate that the population was suitable for QTL analysis of the two traits.

### Quantitative trait locis of crude fiber and crude protein contents

**FIGURE 2:**
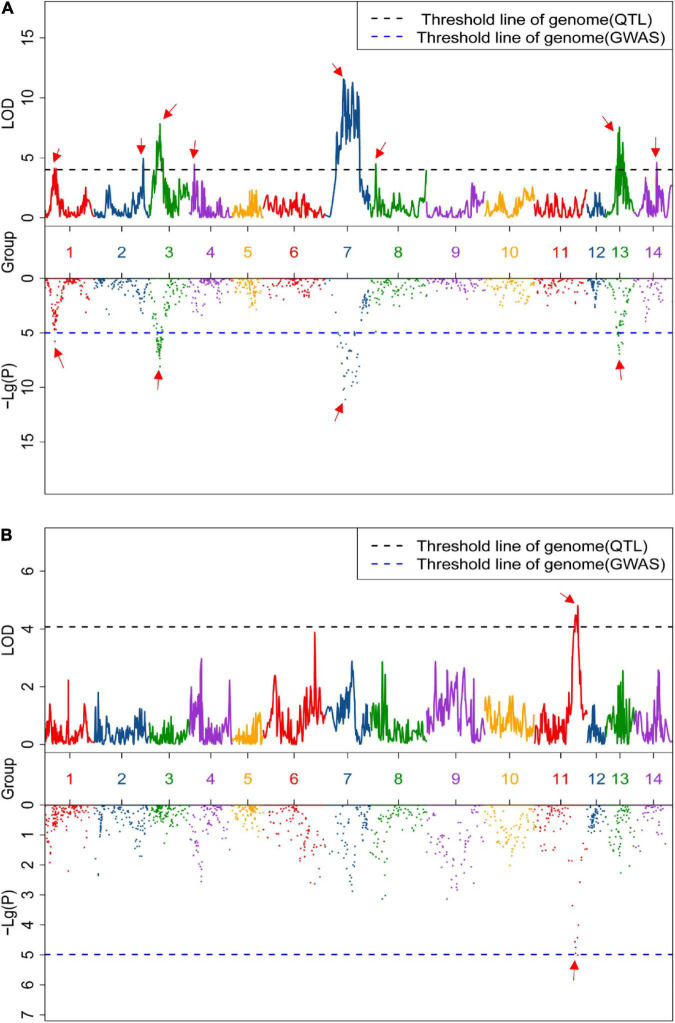
Quantitative trait loci (QTLs) of the content of crude fiber **(A)** and crude protein **(B)**. Upper and lower box indicates the genome-wide LOD value from linkage mapping and the Manhattan plot from association analysis, respectively. Black and blue dashed lines represent the significance threshold value, and the red arrow indicates the significant peak of the QTLs.

Linkage and association mapping methods were used for QTL analysis based on the 1,085 SNPs identified in this F_2_ population. For linkage mapping analysis, the likelihood of odd (LOD) threshold values were estimated using a permutation test program, resulting in 4.05 and 4.36 threshold values used to detect QTLs for crude protein and fiber content, respectively. Consequently, eight QTLs were identified for crude fiber content, accounting for 92.7% of the total phenotypic variation in the population (Table 1 and Figure 2A), and one QTL for crude protein content was detected (Figure 2B). Of these nine QTLs from the linkage mapping method, five could be further validated by the association mapping method (*p* < 1 × 10^–5^), including the crude protein content QTL *CP11* that was flanked by Marker_5028 and Marker_5652 on chromosome 11 (Table 1). Notably, a QTL on chromosome 7 (*CF7*) had a large effect on the trait, with a LOD of 11.57, accounting for 19.75% of the variation within the population. Furthermore, we identified 34 SNPs from the *CF7* region that were significantly associated with crude fiber content (*p* < 1 × 10^–5^), especially Marker_6117 (position: 1,497,478 nt of PHNS01007451.1) with a *p*-value of 6.73 × 10^–14^ (Supplementary Table 1). Additionally, the QTL from chromosome13 (*CF13*) had a large effect on crude fiber, and Marker_75 (position: 458,905 nt of PHNS01003683.1) in this QTL region was significantly associated with the crude fiber content of leaves, with a *p*-value of 5.79 × 10^–11^. These results indicate that *CF7* and *CF13* are two major loci for crude fiber content.

**TABLE 1:** QTLs for the crude fiber and crude protein content detected in the F_2_ population derived from cultivated ZSZ1 and wild tenacissima.

Trait	QTL	Chromosome	Linkage mapping				Association mapping		
			LOD peak	Interval	LOD value	PVE%*^a^*	Peak position	Peak signal	*P*-value
Crude fiber	*CF1*	1	31.5	Marker_3658-Marker_3355	4.11	8.15	34.1	Marker_5183	5.84E-07
	*CF2*	2	165.7	Marker_5166-Marker_3138	4.95	9.65			
	*CF3*	3	36.4	Marker_4874-Marker_4926	7.84	14.45	34.0	Marker_3004	1.8E-09
	*CF4*	4	16.2	Marker_5348-Marker_1230	4.47	8.85			
	*CF7*	7	62.4	Marker_3213-Marker_1520	11.57	19.75	64.7	Marker_6117	6.73E-14
	*CF8*	8	19.2	Marker_5524-Marker_234	4.48	8.85			
	*CF13*	13	46.4	Marker_1765-Marker_5977	7.54	13.95	46.1	Marker_75	5.79E-11
	*CF14*	14	76.7	Marker_2046-Marker_3174	4.61	9.05			
Crude protein	*CP11*	11	146.2	Marker_5028-Marker_5652	4.81	18.1	145.2	Marker_5028	9.75E-06

^a^Percentage of total phenotypic variance explained by the QTL.

### Physical intervals of *CF7* and *CF13*

**FIGURE 3:**
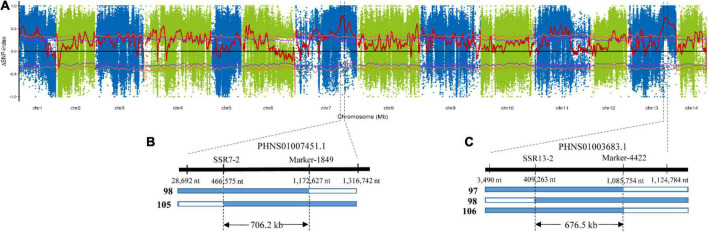
Physical intervals of two major QTLs of crude fiber content, *CF7* and *CF13*. **(A)** ΔSNP-index was calculated from the difference between the SNP-indexes of the pooled bulks. Red lines indicate the ΔSNP-index. Purple and orange lines represent 95 and 99% confidence levels, respectively. In total, two and three recombinant individuals from 30 F_2_ progenies with extremely low crude fiber content delimited the *CF7* **(B)** and *CF13* **(C)** into exact physical intervals, respectively. Other F_2_ individuals with extremely low crude fiber content are not recombinant, and only tenacissima allele was observed in the corresponding QTL regions. Then, two outer numbers under the thick line represent the position of SNPs delimited the region of QTL from BSA, and the intermediated number under the thick line indicates the position of markers used for genotyping the recombinant individuals.

To identify the exact genomic regions of *CF7* and *CF13*, bulked segregant analysis (BSA) was performed using 30 F_2_ individuals with extremely high (>14%) and low (<7%) leaf crude fiber content. BSA sequencing for two pools and their parents, ZSZ1 and tenacissima, generated 583.3 million clean reads (Table 2). After mapping to the ramie reference genome, approximately 103.4–131.1 million reads were aligned with the reference, resulting in an average alignment rate of 88.2% with an average depth of 48.3-folds. Consequently, 6,601,156 and 4,577,790 SNPs were identified in the pool of individuals with extremely high and low crude fibers, respectively. The ΔSNP-index algorithm was used to calculate the allele segregation of the SNPs between the two extreme pools, resulting in two candidate regions in accordance with the QTL regions of *CF7* and *CF13* (Figure 3A). Investigation into the location of SNPs with a ΔSNP-index > 0.8 indicated that these SNPs were in the contigs PHNS01007451.1 (from 28,692 to 1,316,742 nt; peak position: 868,229 nt) and PHNS01003683.1 (from 3,490 to 1,124,784 nt; peak position: 556,306 nt), respectively. Furthermore, in these two intervals determined from BSA, 19 SSR markers were developed and were used to further delimit the QTLs. Finally, *CF7* and *CF13* were mapped into a physical interval of PHNS01007451.1 (from 466,575 to 1,172,627 nt) and PHNS01003683.1 (from 409,263 to 1,085,754 nt), with a length of 706.2 and 676.5 kb, respectively (Figures 3B,C).

**TABLE 2:** Summary of data from BSA sequencing.

Sample	Clean reads	Mapped reads	Mapped rate (%)	Sequencing depth	1X_coverage (%)	5X_coverage (%)	SNPs	Indel
Tenacissima	123,725,880	103,386,701	89.12	41.3035	86.04	77.68	5,858,539	1,057,606
ZSZ1	148,700,594	128,653,525	93.05	52.7234	94.86	92.29	1,877,479	526,075
High crude fiber_pool	124,557,484	104,243,080	93.05	43.9282	94.06	88.18	6,601,156	1,244,618
Low crude fiber_pool	186,287,492	131,104,675	77.54	55.1119	91.54	85.05	4,577,790	895,275

### Identification of a fiber growth-related *whole_GLEAN_10016511* in the *CF13* region

**FIGURE 4:**
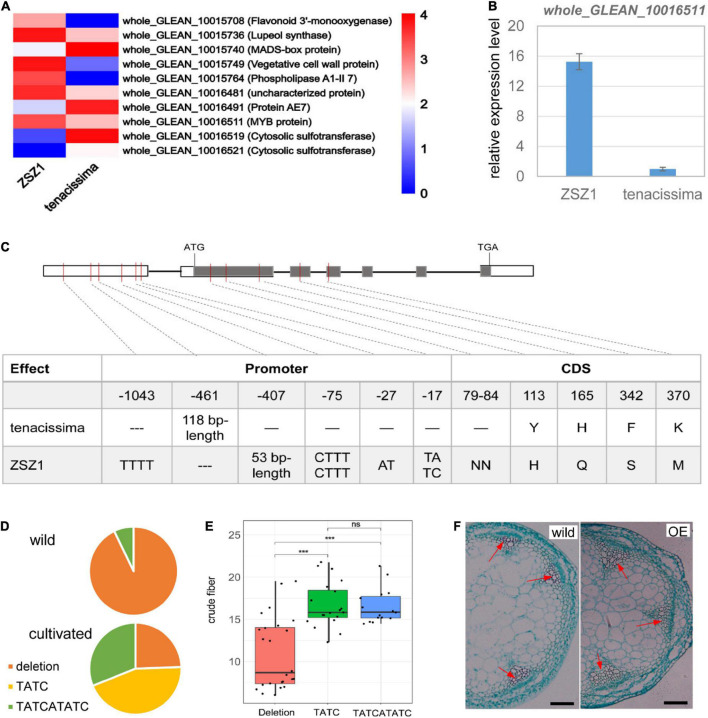
Functional characterization of *whole_GLEAN_10016511*. **(A)** Differentially expressed genes between ZSZ1 and tenacissima leaves. *whole_GLEAN_10015708*, *whole_GLEAN_10015736*, *whole_GLEAN_10015740*, *whole_GLEAN_10015749*, and *whole_GLEAN_10015764* are in the *CF7* region, whereas all others are from the *CF13* region. Gene annotation was indicated in brackets following the gene ID. *whole_GLEAN_10016511* expression was downregulated in the tenacissima leaves. **(B)** Relative expression level of *whole_GLEAN_10016511* in the leaves of ZSZ1 and tenacissima. **(C)** Sequence variations of *whole_GLEAN_10016511* in the two parents, ZSZ1 and tenacissima. Gray and white rectangles indicate the coding sequence (CDS) and promoter region, respectively. The thick black lines before the inter-exon region represent the putative intron region. The vertical molding in the gene indicates variation in the corresponding position of the gene. The tables under each figure show the detailed sequence variations in CDS and the corresponding amino acid change for each gene. **(D)** Allelic variation frequency at the 17-bp upstream of *whole_GLEAN_10016511* CDS in wild and cultivated ramie. Orange, yellow, and green parts in each pie graph indicate the allelic frequency of deletion, insertion by TATC, and insertion by TATCATATC in the wild and cultivated groups. **(E)** Boxplot showed the difference in the crude fiber content of leaves among three groups whose accessions harbor different genotypes at the 17-bp upstream of *whole_GLEAN_10016511* CDS. *** Indicates the significant difference at the level of 0.001, and ns indicates no significant difference. **(F)** Light microscope observation of transected stems of wild and *whole_GLEAN_10016511*-overexpressing (OE) *Arabidopsis*. Arrows indicate the fiber cells of xylem regions. Scale bar = 200 μm.

A total of 82 and 79 predicted genes were included in the interval of *CF7* and *CF13* (Supplementary Tables 2, 3); of these genes, respectively, five from *CF7* and five from *CF13* showed differential expression between the leaves of two parents, ZSZ1 and tenacissima, including an MYB gene *whole_GLEAN_10016511* whose expression level was significantly downregulated in the tenacissima (Figure 4A). MYB transcription factors are crucial for controlling fiber growth by regulating the thickening of the secondary wall (Zhong and Ye, 2015). whole_GLEAN_10016511 is an ortholog of *Arabidopsis* KAN2 (Supplementary Figure 1). KAN2 plays a crucial role in vascular tissue formation, and its loss-of-function mutants cause ectopic xylem and fiber differentiation in amphivasal vascular tissue (Ilegems et al., 2010). Recently, Genome-wide Association Studies (GWAS) analysis identified a significant signal associated with the fiber content of stem barks in ramie, and *whole_GLEAN_10016511* was near this signal (Huang et al., 2021). Therefore, *whole_GLEAN_10016511* might be the candidate of *CF13* and was further examined. We first performed qRT-PCR analysis for *whole_GLEAN_10016511* and further verified its differential expression in the leaves of ZSZ1 and tenacissima (Figure 4B). Then, a sequence comparison for *whole_GLEAN_10016511* was conducted and identified five variations that caused amino acid change between the parents, including a deletion in the tenacissima (Figure 4C). Notably, there were numerous variations in the promoter region of the gene, including a 53-bp deletion and a 118-bp insertion in tenacissima comparing with ZSZ1 (Figure 4C). Similarly, recent resequencing of 46 cultivated and 14 wild germplasms revealed wide variations in the promoter region of *whole_GLEAN_10016511* (Wang et al., 2021). Using the Indels at the 17-bp upstream of CDS as an example, there were three genotypes in 60 accessions, i.e., deletion and insertion by TATC and TATCATATC, respectively. Interestingly, 13 of 14 wild accessions had a genotype with the deletion at this site, whereas among 35 accessions with the insertion genotype, 34 were cultivated (Figure 4D), indicating the selection for this gene during ramie domestication. Importantly, accessions harboring deletion genotype exhibited a significantly lower content of crude fiber in leaves than those harboring the insertion genotype (Figure 4E). Finally, we explored the function of *whole_GLEAN_10016511* by executing an overexpressed analysis in *Arabidopsis*, resulting in a phenotype with more fiber cells observed in the stem of the transgenic plants (Figure 4F and Supplementary Figure 2). Taken together, our results indicated that *whole_GLEAN_10016511* is a fiber growth-related gene, and numerous variations in its promoter region in tenacissima cause low expression levels, likely responsible for the relatively low fiber content of wild tenacissima. Importantly, *whole_GLEAN_10016511* was in the *CF13* region, and *whole_GLEAN_10016511* is a logical candidate for this QTL.

## Discussion

Ramie has a close evolutionary relationship with *Morus alba*, a forage plant that is eaten by silkworms, and their genomes show a high level of collinearity (Wang et al., 2021). Similar to *Morus alba*, ramie leaves have a high crude protein content. A recent study identified 106 ramie genes that underwent positive selection, and 22 of these were enriched in the GO term “nitrogen compound metabolism,” suggesting that the positive selection of these nitrogen metabolism-related genes could be responsible for crude protein biosynthesis in domesticated ramie (Liu et al., 2018). Currently, the genetic basis of the traits that reflect forage quality remains poorly understood. In this study, we identified nine QTLs using the linkage mapping method. Although linkage maps have been proven to be a powerful tool for identifying genetic loci of agronomic traits in plants using biparental primary populations (Liu et al., 2010, 2011), it is still challenging to determine candidate genes from QTL analysis because of its low precision in genetic mapping. To circumvent this problem, two other methods were used alongside QTL mapping. Association mapping was used to verify the findings of linkage mapping and revealed that five of nine QTLs (four and one, for crude fiber and protein, respectively) were reliable. Additionally, BSA sequencing was used to further improve the precision of the location of the two major QTLs for crude fiber content in ramie leaves, thereby making it feasible to identify the candidates for these two QTLs. Taken together, this study provided the first insights into the genetic basis underlying the traits of crude protein and fiber content in ramie, which will allow forage quality improvement using marker-assisted selection.

As a fiber crop, ramie produces numerous fibers in its bast bark; even in its leaves, there are plentiful crude fibers, which leads to poor palatability as forage. Plant fibers include thickened secondary cell walls composed of cellulose, hemicelluloses (xylan and glucomannan), and lignin, which are deposited in some specialized cells (Zhong and Ye, 2015). Biosynthesis of secondary walls is regulated by a NAC-MYB-based transcriptional network (Nakano et al., 2015), and at least 16 MYB proteins are involved in this network in *Arabidopsis* (Zhong and Ye, 2015). Several ramie MYB genes have been identified to be involved in fiber growth. *evm.model.scaffold7373.133_D1* is a candidate for *qBT4a*, a QTL of the bark thickness trait related to fiber yield, and it encodes a putative MYB protein. There was a 760-bp insertion that caused premature termination and produced a protein that lacked a part of the MYB domain (Liu et al., 2017). *whole_GLEAN_10015497* is another MYB gene associated with fiber growth in ramie, and its overexpression caused a distinct increase in the number of fibers in transgenic plants (He et al., 2021). In this study, a fiber growth-promoted MYB gene, *whole_GLEAN_10016511*, was mapped on the *CF13* region, a QTL for crude fiber content. Wide variations in the promoter region of this MYB gene caused its low expression in tenacissima, a parent with low crude fiber content in leaves. These findings supported the proposal that *whole_GLEAN_10016511* is a candidate for *CF13*. These results provided an essential basis for the cloning of *CF13* in the future studies.

Wild tenacissima is the progenitor of cultivated ramie, and many variations have been identified in the genome of tenacissima and cultivated ZSZ1, most of which impact the trait performance. For example, *BntGA2ox1* codes for gibberellin 2-beta-dioxygenase, and an 11.7-kb insertion in this gene causes a genic structural change in the genome of cultivars, which is potentially associated with long stems and fibers in cultivated ramie (Wang et al., 2021). This study identified numerous variations in the fiber growth-promoted *whole_GLEAN_10016511* between tenacissima and ZSZ1 alleles, especially in its promoter region. Of these variations, an Indels at the 17-bp upstream of CDS had a considerable relationship with the crude fiber content of leaves. Variations in the promoter of *whole_GLEAN_10016511* could cause high gene expression levels in ZSZ1 compared to tenacissima and are likely responsible for the high content of crude fiber of cultivated ZSZ1 leaves. Therefore, breeding selection using the tenacissima allele of *whole_GLEAN_10016511* could theoretically decrease the crude fiber content, thereby improving ramie palatability.

## Materials and methods

### Experimental material and field planting

An existing F_2_ population consisting of 111 progenies derived from cultivated ZSZ1 and wild tenacissima was used for linkage mapping analysis (Supplementary Figure 3; Wang et al., 2019). Cuttings from propagations of each F_2_ individual and the parents were taken, and five seedlings of each line were grown in the experimental farm at the Institute of Bast Fiber Crops (28.50°N, 112.22°E), Chinese Academy of Agricultural Sciences, Yuanjiang, China, in 2017. In April 2018, young leaves were collected, dried, and used to estimate the crude protein and fiber content.

### Phenotype measurements

Crude fiber content was determined using the intermediate filtration method (ISO 6865:2000, 2000). Briefly, 1 g of dried leaves was pre-degreased using petroleum ether, and carbonate content was removed using hydrochloric acid (0.5 mol/L). Subsequently, the treated leaves were boiled in a sulfuric acid solution (0.13 mol/L) for 30 min. After degreasing with petroleum ether, the sample was boiled in a potassium hydroxide solution (0.23 mol/L) for 30 min. After filtering and drying, the samples were weighed (m1). Finally, the samples were subjected to be ashed and weighed again (m2). The difference between m1 and m2 was used to estimate crude fiber, which was further used to calculate the crude fiber content of dried leaves (%).

The Kjeldahl method was used to estimate the crude protein content (CNS GB/T 6432-2018, 2018). Briefly, 0.5 g of dried leaves was boiled in a 12 ml of sulfuric acid solution for 60 min and catalyzed using a mixture of copper sulfate (0.4 g) and sodium sulfate (6 g). Thereafter, the nitrogen compounds in the sample were resolved in a sodium hydroxide solution (0.4 g/ml), and the generated ammonia was collected using a boric acid solution (20 mg/ml). Finally, the ammonia content in the boric acid solution was estimated using titration with hydrochloric acid (0.02 mol/L), which was used to determine the crude protein content.

The correlation between the crude protein and fiber content was estimated using SPSS software, and significance was set at *p* ≤ 0.05.

### Quantitative trait loci analysis

Based on the linkage map of the F_2_ population (Wang et al., 2019), QTLs for the two traits examined were detected using the QTL ICIMAPPING (v4.2) program (Meng et al., 2015). The experiment-wise LOD threshold significance level was determined by computing 1,000 permutations (*p* < 0.05) using a permutation test program. To perform associated mapping, all 1,085 SNPs in the linkage map were used for association analysis with trait phenotypes using the EMMAX program (Kang et al., 2010), and a *p-value* threshold for the suggested locus associations was set to *p* < 1 × 10^–5^.

### Bulked segregant analysis sequencing

Another population consisting of 412 F_2_ individuals from the crossing of ZSZ1 and wild tenacissima was used (Supplementary Figure 3), and the content of crude fiber in leaves of these 412 individuals was determined. In total, thirty individuals with > 14% crude fiber in the leaves were pooled as a sample, whereas 30 individuals with < 7% crude fiber in leaves were used as another pooled sample. Genomic DNAs of these two pooled samples together with two parents were separately extracted from the leaves using a DNA Extraction Kit (TIANGEN Biotech, Beijing, China) according to the manufacturer’s instructions. They were used to construct a sequencing library with the TruSeq Nano Sample Prep Kit (Illumina Inc., San Diego, CA, United States) according to the manufacturer’s specifications. Subsequently, sequencing was performed using the Illumina Hiseq X platform.

After filtering the low-quality reads, the clean reads were aligned to the reference genome of ramie (variety: Zhongzhu NO. 1),^1^ using Burrows–Wheeler Aligner (BWA) software (v.0.7.8) (Li and Durbin, 2009), with the default parameters. The alignment results were then converted into BAM format and sorted using the SAMtools (v.1.3) (Li et al., 2009). Subsequently, SNPs and Indels for each sample were identified using the Bayesian approach implemented in the package SAMtools. SNPs with a different and homozygous allele in two parents were further filtered using GATK software (McKenna et al., 2010). The SNP-index represents the ratio of reads harboring SNPs among the entire number of reads (Abe et al., 2012), and the ΔSNP-index was the difference of the SNP-index between pooled bulks. To identify the candidate regions associated with crude fiber content, the ΔSNP-index of each locus was calculated by subtracting the SNP-index of the low crude fiber pool from that of the high crude fiber pool according to the previous method (Takagi et al., 2013).

### Simple sequence repeat markers analysis

The SSRs were detected in the target regions using the software of AutoSSR (Wang et al., 2009), with the default parameters. In total, 30 individuals with an extremely low crude fiber of leaves (<7%) from 412 F_2_ progenies were individually extracted genomic DNAs. The SSRs were performed for PCR amplification using the primer pairs in Supplementary Table 4. The SSR assay was conducted as described by Wu and Tanksley (1993).

### Expression analysis

RNA sequencing for the leaves of ZSZ1 and tenacissima had been completed by our previous study (Wang et al., 2021). Fragments per kilobase per million read values of genes fell into the QTL regions were collected from these reported transcriptome data and were used to compare their expression level between two varieties. Heatmap of gene expression was visualized using an online tool.^2^ Total RNAs of young leaves sampled from ZSZ1 and tenacissima were extracted, reverse-transcribed, and used for qRT-PCR analysis. Briefly, qRT-PCR was performed using iTaq™ Universal SYBR Green SuperMix (Bio-Rad, United States) using an optical 96-well plate with an iQ5 multicolor real-time PCR system (Bio-Rad). The 18S ribosomal RNA gene was used as an internal control. The primer sequences are listed in Supplementary Table 4. The relative expression level was determined according to the method proposed by Livak and Schmittgen (2001).

### Sequence comparison and overexpression for *whole_GLEAN_10016511*

Genomic DNAs of ZSZ1 and tenacissima were used to amplify the genic region and its upstream 1.5-kb sequence for *whole_GLEAN_10016511* using a standard PCR protocol with gene-specific primers (Supplementary Table 4). After being digested using 5 U of ExoI (NEB) and 0.13 U of shrimp alkaline phosphatase (Fermentas), the PCR products were performed for Sanger sequencing using a 3730xl DNA Analyzer (ABI, United States). The sequence contigs were assembled using SEQUENCER 4.1.2 (Gene Codes Co.), and the assembled sequences were aligned and compared using Clustal Omega (Sievers et al., 2011).

To clone the candidate of *whole_GLEAN_10016511*, the full-length sequence of this gene was amplified from a cDNA library by a high-fidelity thermostable DNA polymerase and specific primer pair (Supplementary Table 4). Then, the amplified sequence was ligated into the PBI121 vector to initiate its expression by the CaMV 35S promoter. The plasmid construct was introduced into *Agrobacterium tumefaciens strain* GV3101 using the heat shock method, and the resulting Agrobacterium was introduced into *Arabidopsis* using the floral dip method (Zhang et al., 2006). Transgenic plants were grown in a greenhouse under the temperature of 22°C and photoperiod of 15-h light/9-h dark cycle. Section of 40-day-old transgenic plants was conducted and stained with Safranin O-Fast Green, which was used to examine the stem cells through transmission light microscopy.

## Data availability statement

The original contributions presented in this study are publicly available. This data can be found here: NCBI, PRJNA842875.

## Author contributions

ZL and ZZ collected the data and performed the experimental laboratory works. SZ performed the data analysis. YW performed the field experiment. XY supervised the work. YW and TL conceived the work and wrote the manuscript. All authors have read and agreed to the published version of the manuscript.

## Footnotes

1
www.ncbi.nlm.nih.gov/assembly/GCA_002937015.1

2
https://cloud.oebiotech.cn/task/detail/heatmap/

## References

[B1] Abe A. Kosugi S. Yoshida K. Natsume S. Takagi H. Kanzaki H. (2012). Genome sequencing reveals agronomically important loci in rice using MutMap. *Nat. Biotechnol.* 30 174–178. 10.1038/nbt.2095 22267009

[B2] Bai X. Wang X. Wang Y. Wei Y. Fu Y. Rao J. (2022). Genome-wide association study of six forage traits in ramie (*Boehmeria nivea* L. Gaud). *Plants* 11:1443. 10.3390/plants11111443 35684216 PMC9182863

[B3] Chen J. Rao J. Wang Y. Zeng Z. Liu F. Tang Y. (2019). Integration of quantitative trait loci mapping and expression profiling analysis to identify genes potentially involved in ramie fiber lignin biosynthesis. *Genes* 10:842. 10.3390/genes10110842 31653111 PMC6896145

[B4] Chen J. Yu R. Liu L. Wang B. Peng D. (2016). Large-scale developing of simple sequence repeat markers and probing its correlation with ramie (*Boehmeria nivea* L.) fiber quality. *Mol. Genet. Genomics* 291 753–761. 10.1007/s00438-015-1143-2 26577947

[B5] Chen K. Luan M. Xiong H. Chen P. Chen J. Gao G. (2018). Genome-wide association study discovered favorable single nucleotide polymorphisms and candidate genes associated with ramet number in ramie (*Boehmeria nivea* L.). *BMC Plant Biol.* 18:345. 10.1186/s12870-018-1573-1 30541445 PMC6292125

[B6] CNS GB/T 6432-2018 (2018). *Determination of crude protein in feeds—Kjeldahl method.* Beijing: National Standardization Administration.

[B7] He Q. Zeng Z. Li F. Huang R. Wang Y. Liu T. (2021). Ubiquitylome analysis reveals the involvement of ubiquitination in the bast fiber growth of ramie. *Planta* 254:1. 10.1007/s00425-021-03652-x 34081200

[B8] Huang K. Shi Y. Pan G. Zhong Y. Sun Z. Niu J. (2021). Genome-wide association analysis of fiber fineness and yield in ramie (*Boehmeria nivea*) using SLAF-seq. *Euphytica* 217:22. 10.1007/s10681-020-02757-w

[B9] Ilegems M. Douet V. Meylan-Bettex M. Uyttewaal M. Brand L. Bowman J. L. (2010). Interplay of auxin, KANADI and class III HD-ZIP transcription factors in vascular tissue formation. *Development* 137 975–984. 10.1242/dev.047662 20179097

[B10] INFIC (1978). *Data from International Network of Feed Information Centres.* Rome: FAO.

[B11] ISO 6865:2000 (2000). *Animal feeding stuffs—determination of crude fibre content—method with intermediate filtration.* Geneva: ISO.

[B12] Kang H. M. Sul J. H. Service S. K. Zaitlen N. Kong S. Freimer N. (2010). Variance component model to account for sample structure in genome-wide association studies. *Nat. Genet.* 42 348–354. 10.1038/ng.548 20208533 PMC3092069

[B13] Li H. Durbin R. (2009). Fast and accurate short read alignment with Burrows-Wheeler transform. *Bioinformatics* 25 1754–1760. 10.1093/bioinformatics/btp324 19451168 PMC2705234

[B14] Li H. Handsaker B. Wysoker A. Fennell T. Ruan J. Homer N. (2009). The sequence alignment/map format and SAMtools. *Bioinformatics* 25 2078–2079. 10.1093/bioinformatics/btp352 19505943 PMC2723002

[B15] Li H. L. (1970). The origin of cultivated plants in Southeast Asia. *Econ. Bot.* 24 3–19. 10.1007/BF02860628

[B16] Liu C. Zeng L. Zhu S. Wu L. Wang Y. Tang S. (2018). Draft genome analysis provides insights into the fiber yield, crude protein biosynthesis, and vegetative growth of domesticated ramie (*Boehmeria nivea* L. Gaud). *DNA Res.* 25 173–181. 10.1093/dnares/dsx047 29149285 PMC5909428

[B17] Liu C. Zhu S. Tang S. Wang H. Zheng X. Chen X. (2017). QTL analysis of four main stem bark traits using a GBS-SNP-based high-density genetic map in ramie. *Sci. Rep.* 7:13458. 10.1038/s41598-017-13762-w 29044147 PMC5647422

[B18] Liu T. Li L. Zhang Y. Xu C. Li X. Xing Y. (2011). Comparison of quantitative trait loci for rice yield, panicle length and spikelet density across three connected populations. *J. Genet.* 90 377–382. 10.1007/s12041-011-0083-9 21869494

[B19] Liu T. Zhang Y. Xue W. Xu C. Li X. Xing Y. (2010). Comparison of quantitative trait loci for 1,000-grain weight and spikelets per panicle across three connected rice populations. *Euphytica* 175 383–394. 10.1007/s10681-010-0186-z

[B20] Liu T. Zhu S. Fu L. Tang Q. Yu Y. Chen P. (2013). Development and characterization of 1,827 expressed sequence tag-derived simple sequence repeat markers for ramie (*Boehmeria nivea* L. Gaud). *PLoS One* 8:e60346. 10.1371/journal.pone.0060346 23565230 PMC3614921

[B21] Liu T. Zhu S. Tang Q. Tang S. (2014). QTL mapping for fiber yield-related traits by constructing the first genetic linkage map in ramie (*Boehmeria nivea* L. Gaud). *Mol. Breed.* 34 883–892. 10.1007/s11032-014-0082-7

[B22] Livak K. J. Schmittgen T. D. (2001). Analysis of relative gene expression data using real-time quantitative PCR and the 2- ΔΔCT method. *Methods* 25 402–408. 10.1006/meth.2001.1262 11846609

[B23] Luan M. Liu C. Wang X. Y. Sun Z. Chen J. (2017). SSR markers associated with fiber yield traits in ramie (*Boehmeria nivea* L. Gaudich). *Ind. Crops Prod.* 107 439–445. 10.1016/j.indcrop.2017.05.065

[B24] McKenna A. Hanna M. Banks E. Sivachenko A. Cibulskis K. Kernytsky A. (2010). The genome analysis toolkit: A MapReduce framework for analyzing next-generation DNA sequencing data. *Genome Res.* 20 1297–1303. 10.1101/gr.107524.110 20644199 PMC2928508

[B25] Meng L. Li H. Zhang L. Wang J. (2015). QTL IciMapping: Integrated software for genetic linkage map construction and quantitative trait locus mapping in biparental populations. *Crop J.* 3 269–283. 10.1016/j.cj.2015.01.001

[B26] Nakano Y. Yamaguchi M. Endo H. Rejab N. A. Ohtani M. (2015). NAC-MYB-based transcriptional regulation of secondary cell wall biosynthesis in land plants. *Front. Plant Sci.* 6:288. 10.3389/fpls.2015.00288 25999964 PMC4419676

[B27] Pérez A. Wencomo H. B. Armengol N. Reyes F. (2013). *Boehmeria nivea* (L.) Gaud. *Pastos Forrajes* 36 404–408.

[B28] Rehman M. Gang D. Liua Q. Chen Y. Wang B. Peng D. (2019). Ramie, a multipurpose crop: Potential applications, constraints and improvement strategies. *Ind. Crops Prod.* 137 300–307. 10.1016/j.indcrop.2019.05.029

[B29] Sievers F. Wilm A. Dineen D. Gibson T. J. Karplus K. Li W. (2011). Fast, scalable generation of high-quality protein multiple sequence alignments using clustal omega. *Mol. Syst. Biol.* 7:539. 10.1038/msb.2011.75 21988835 PMC3261699

[B30] Subandi M. (2012). The effect of fertilizers on the growth and the yield of ramie (*Boehmeria nivea* L. Gaud). *Asian J. Agric. Rural Dev.* 2 126–135.

[B31] Takagi H. Abe A. Yoshida K. Kosugi S. Natsume S. Mitsuoka C. (2013). QTL-seq: Rapid mapping of quantitative trait loci in rice by whole genome resequencing of DNA from two bulked populations. *Plant J.* 74 174–183. 10.1111/tpj.12105 23289725

[B32] Wang C. Guo W. Zhang T. Li Y. Liu H. (2009). AutoSSR: An improved automatic software for SSR analysis from large-scale EST sequences. *Cotton Sci.* 2l 243–247.

[B33] Wang Y. Li F. He Q. Bao Z. Zeng Z. An D. (2021). Genomic analyses provide comprehensive insights into the domestication of bast fiber crop ramie (*Boehmeria nivea*). *Plant J.* 107 787–800. 10.1111/tpj.15346 33993558

[B34] Wang Y. Zeng Z. Li F. Yang X. Gao X. Ma Y. (2019). A genomic resource derived from the integration of genome sequences, expressed transcripts and genetic markers in ramie. *BMC Genomics* 20:476. 10.1186/s12864-019-5878-8 31185891 PMC6558782

[B35] Wu K. S. Tanksley S. D. (1993). Abundance, polymorphism and genetic mapping of microsatellites in rice. *Mol. Gen. Genet.* 241 225–235. 10.1007/BF00280220 7901751

[B36] Zeng Z. Wang Y. Liu C. Yang X. Wang H. Li F. (2019). Linkage mapping of quantitative trait loci for fiber yield and its related traits in the population derived from cultivated ramie and wild *B. nivea* var. *tenacissima*. *Sci. Rep.* 9:16855. 10.1038/s41598-019-53399-5 31728008 PMC6856109

[B37] Zeng Z. Zhu S. Wang Y. Bai X. Liu C. Chen J. (2022). Resequencing of 301 ramie accessions identifies genetic loci and breeding selection for fiber yield traits. *Plant Biotechnol. J.* 20 323–334. 10.1111/pbi.13714 34558775 PMC8753365

[B38] Zhang X. Henriques R. Lin S. S. Niu Q. W. Chua N. H. (2006). Agrobacterium-mediated transformation of *Arabidopsis thaliana* using the floral dip method. *Nat. Protoc.* 1 641–646. 10.1038/nprot.2006.97 17406292

[B39] Zhong R. Ye Z. H. (2015). Secondary cell walls: Biosynthesis, patterned deposition and transcriptional regulation. *Plant Cell Physiol.* 56 195–214. 10.1093/pcp/pcu140 25294860

[B40] Zhu S. Zheng X. Dai Q. Tang S. Liu T. (2016). Identification of quantitative trait loci for flowering time traits in ramie (*Boehmeria nivea* L. Gaud). *Euphytica* 210 367–374. 10.1007/s10681-016-1692-4

