# Supplementary material for: Genetic insights into the crude protein and fiber content of ramie leaves
Source: Front Plant Sci. 2022 Oct 4;13:969820. doi: 10.3389/fpls.2022.969820 (PMC9577236; doi:10.3389/fpls.2022.969820)
Supplement: Supplementary file 1 [file Data_Sheet_1.docx]

**Supplementary Fig S1** Phylogenetic tree of whole_GLEAN_10016511 and Arabidopsis fiber growth-related MYB protein.


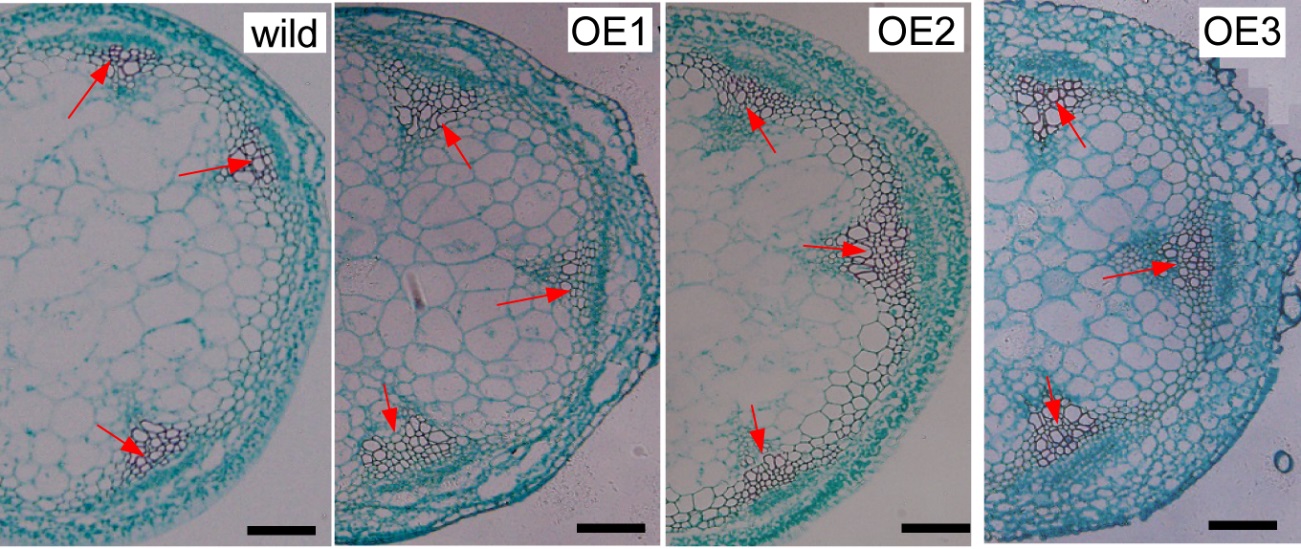


**Supplementary Fig S2** Microscope observation of transected stems of wild and *whole_GLEAN_10016511*-overexpressing (OE) Arabidopsis. Arrows indicate the fiber cells of xylem regions. Scale bar = 200 μm.


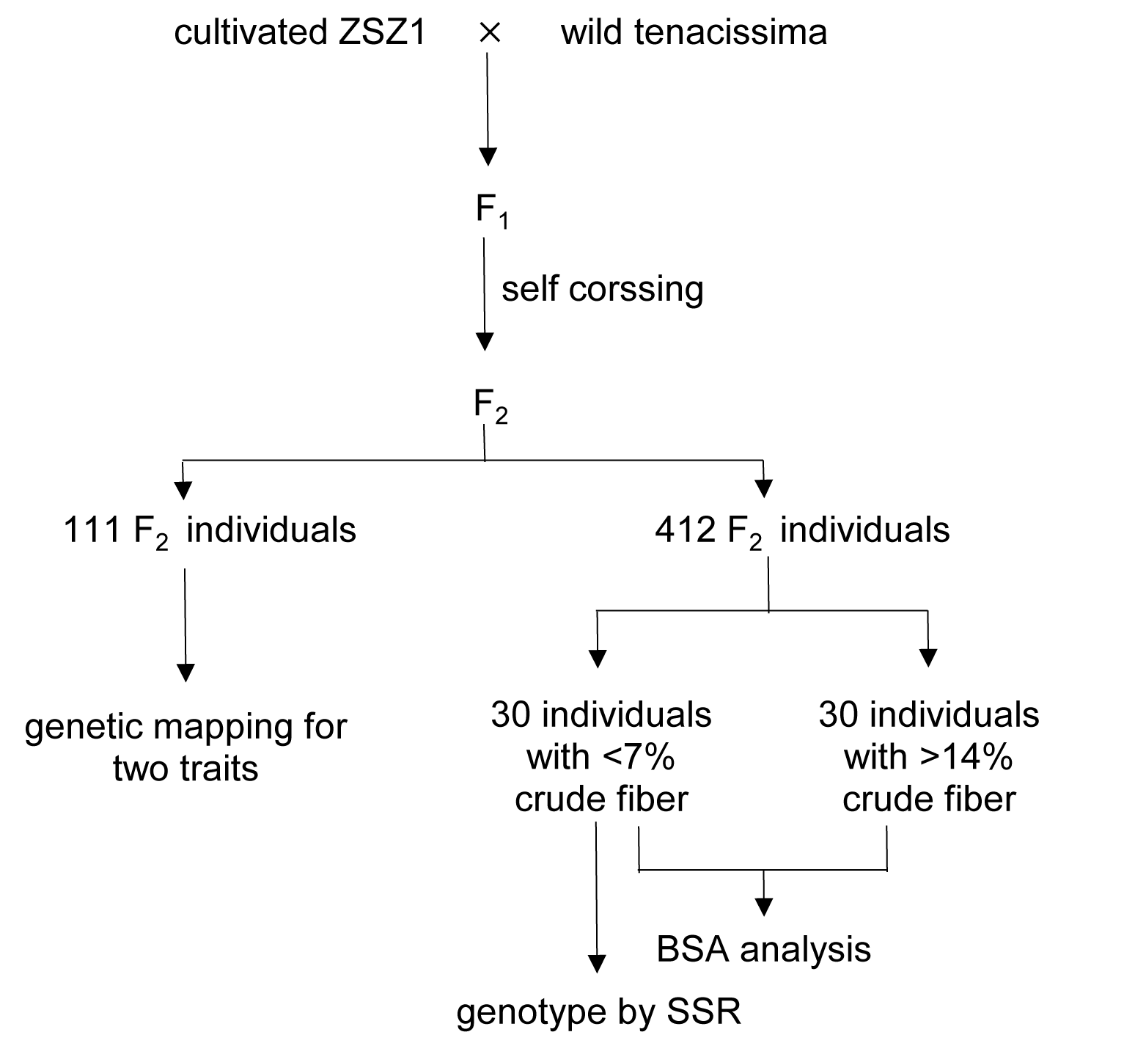


**Supplementary Fig S3** Flow chart showed the construction of experimental material.

**Supplementary Table S1** Markers that showed significant association with the content of crude fiber in *CF7* region

| Marker | Genetic location (cM) | P value from the association analysis |
| --- | --- | --- |
| Maker_6112 | 41.711 | 1.18E-08 |
| Maker_3517 | 43.254 | 4.64E-08 |
| Maker_5873 | 50.825 | 2.90E-09 |
| Maker_6114 | 53.587 | 1.34E-09 |
| Maker_5586 | 56.923 | 3.52E-09 |
| Maker_1845 | 59.098 | 2.88E-10 |
| Maker_3213 | 61.378 | 2.83E-12 |
| Maker_1520 | 63.36 | 1.13E-12 |
| Maker_6117 | 64.669 | 6.73E-14 |
| Maker_1522 | 66.405 | 3.31E-09 |
| Maker_3669 | 68.404 | 3.95E-11 |
| Maker_6116 | 71.16 | 1.26E-07 |
| Maker_1371 | 72.986 | 3.48E-09 |
| Maker_1856 | 74.667 | 6.29E-10 |
| Maker_1849 | 75.543 | 1.82E-10 |
| Maker_1345 | 78.378 | 6.28E-13 |
| Maker_1346 | 79.147 | 3.30E-15 |
| Maker_1348 | 80.424 | 3.67E-13 |
| Maker_1351 | 83.896 | 4.26E-10 |
| Maker_1350 | 84.525 | 2.03E-10 |
| Maker_1478 | 89.095 | 1.06E-09 |
| Maker_1147 | 90.102 | 7.81E-14 |
| Maker_4784 | 92.565 | 2.81E-11 |
| Maker_1146 | 94.08 | 4.07E-13 |
| Maker_249 | 98.886 | 2.14E-08 |
| Maker_250 | 99.297 | 3.51E-08 |
| Maker_252 | 100.655 | 6.54E-10 |
| Maker_251 | 101.014 | 4.53E-09 |
| Maker_5783 | 104.53 | 8.42E-08 |
| Maker_5786 | 106.976 | 3.30E-08 |
| Maker_5784 | 108.348 | 1.97E-07 |
| Maker_5787 | 109.062 | 2.63E-09 |
| Maker_4916 | 110.714 | 3.58E-08 |
| Maker_29 | 115.465 | 6.22E-06 |

**Supplementary Table S4** Primer sequences for PCR amplification

| Purpose | Primer ID |  | Sequence |
| --- | --- | --- | --- |
| SSRs in the *CF7* and *CF13* region | SSR7-1 | Forward primer | CGAGACCAACCAGGAATTACGT |
|  |  | Reverse primer | ACGGTAACGAAAACAAACAGAGC |
|  | SSR7-2 | Forward primer | GGTTTATGGCGAACTGTGAC |
|  |  | Reverse primer | ACGAGGCTGGCTTCTCTGTAG |
|  | SSR7-3 | Forward primer | AGTTGGTGTCGATCAGGCAGT |
|  |  | Reverse primer | AGTTGGTGTCGATCAGGCAGT |
|  | SSR7-4 | Forward primer | TGCCTCTAATTGTTGCAGTTCC |
|  |  | Reverse primer | TCTGGTCATTTTATCTAGTATGGTGTG |
|  | SSR7-5 | Forward primer | TTATGGTTCTTATCCGAAATTGTG |
|  |  | Reverse primer | TGCTTTAGATGATGGCTTGGTT |
|  | SSR7-6 | Forward primer | TATGCTCCCCTGATTCGTGAT |
|  |  | Reverse primer | TGATTAGTCCATCCATCTGGGTTA |
|  | SSR7-7 | Forward primer | AGCACTTCGACGTCCTCACT |
|  |  | Reverse primer | GCATTCAAAACCTCAACATCC |
|  | SSR7-8 | Forward primer | AGTTCCTAAAACAACCCCTCCAT |
|  |  | Reverse primer | GCACTTCCAACCCTCTTCTCAT |
|  | SSR7-9 | Forward primer | ATGTTGACCTGATGACTATTTTGCT |
|  |  | Reverse primer | GTGACCAAAGCCATAAACATAGAAC |
|  | SSR7-10 | Forward primer | GCACTTCCAACCCTCTTCTCAT |
|  |  | Reverse primer | GCAAAGTCATGCATTGATCAAAGT |
|  | SSR13-1 | Forward primer | AGGATGTGTTTGCTCATTTGG |
|  |  | Reverse primer | GAGGACTACTTGTTTTCCTCCA |
|  | SSR13-2 | Forward primer | CGCGTATTGTAGCCATCTCTTG |
|  |  | Reverse primer | ACGGTTCTAGCCATGGAATCTT |
|  | SSR13-3 | Forward primer | TCAGGATGCAAAACAAGTCGAA |
|  |  | Reverse primer | GCTTTCCTTTTACTGCAGCTTCT |
|  | SSR13-4 | Forward primer | TTCGCCGGTGAGAGAGGTTT |
|  |  | Reverse primer | TCTCCTCTTTGCTCTTTTTCGGT |
|  | SSR13-5 | Forward primer | GGGGACTAAATGAAACAAGAGAT |
|  |  | Reverse primer | CGTATGTATGTGCATGTTCCTGAT |
|  | SSR13-6 | Forward primer | GAGAGAGAGAGAAATAAGTGGAGC |
|  |  | Reverse primer | TGCATGGTTCGAGGAAATATAA |
|  | SSR13-7 | Forward primer | TCATTCATTCATTCGCTCACAT |
|  |  | Reverse primer | GGTGTGATGTAAATGTATTGGCT |
|  | SSR13-8 | Forward primer | GCTCTAAAGTTCCAGTCAGAAAAAT |
|  |  | Reverse primer | ACGGAAACAACAAGGTCGAGA |
|  | SSR13-9 | Forward primer | GGAGAGAACAGCCATGGTCAA |
|  |  | Reverse primer | CCGATATTACTGCGCTGTTACTT |
| Sequence comparison of *whole_GLEAN_10016511* | 11-1 | Forward primer | CAGGTTCTTGCTCCTTTCTTT |
|  |  | Reverse primer | CTGCATATGTGTGTGTATGGG |
|  | 11-2 | Forward primer | AAATAGAAAGAGGTTTGGGAATC |
|  |  | Reverse primer | TATAATCTGCCTCTTAGTGGGTT |
|  | 11-3 | Forward primer | CTTTGAGACTAAGTTTTGGTATTCC |
|  |  | Reverse primer | TCGGACGGTTAAGACTACAGATA |
|  | 11-4 | Forward primer | CACTTGACCGAATTCCCTG |
|  |  | Reverse primer | TACCCATTTTTGCTATCCTCA |
|  | 11-5 | Forward primer | TTATACAACTCTGGGTCGTAGTGT |
|  |  | Reverse primer | CTTCTACCACTAATGCCAACAAC |
|  | 11-6 | Forward primer | TTGGTGGTGGTGGTGAAGCT |
|  |  | Reverse primer | CAAAATCTGCAACTACTCCCTC |
|  | 11-7 | Forward primer | TTTGCTTCTTTTATGTGGTAAGG |
|  |  | Reverse primer | TCTATGATTCAAATGGGCAAGT |
| qRT-PCR | *whole_GLEAN_10016511* | Forward primer | GGCTCGACGTGTTCCTCGAG |
|  |  | Reverse primer | AGATGTCCGACGCCTTGTCC |
|  | 18s | Forward primer | AGACTGTGAAACTGCGAATG |
|  |  | Reverse primer | AATCATCTGAGCAACGGG |
| Overexpression of *whole_GLEAN_10016511* | | Forward primer | ATGGAGCTATTTCCTGCTCAAC |
|  |  | Reverse primer | AAGTGCCCTTCCCAAAGTGA |
